# Patient Perspectives on Evolving Diverticulitis Treatment: An Assessment of Patient Willingness to Enroll in a Randomized Controlled Trial

**DOI:** 10.1097/AS9.0000000000000476

**Published:** 2024-09-05

**Authors:** Annie Altman-Merino, Kemberlee Bonnet, David Schlundt, Jesse Wrenn, Wesley H. Self, Elisa J. Gordon, Alexander T. Hawkins

**Affiliations:** From the * Department of School of Medicine, Vanderbilt University, Nashville, TN; † Department of Psychology, Vanderbilt University, Nashville, TN; ‡ Department of Emergency Medicine, Vanderbilt University Medical Center, Nashville, TN; § Department of Emergency Medicine, Vanderbilt University Medical Center, Vanderbilt Institute for Clinical and Translational Research, Nashville, TN; ‖ Department of Surgery, and Center for Biomedical Ethics and Society, Vanderbilt University Medical Center, Nashville, TN; ¶ Department of Surgery, Section of Colon & Rectal Surgery, Vanderbilt University Medical Center, Nashville, TN.

## Abstract

**Objective:**

The objective of the study was to assess patients’ attitudes regarding participation in a randomized trial of antibiotics *versus* placebo for acute diverticulitis.

**Background:**

Despite evidence that antibiotics may not be necessary to treat acute uncomplicated diverticulitis, they remain the mainstay of treatment in the United States. A randomized trial in the United States evaluating antibiotic effectiveness could accelerate the implementation of antibiotic-free treatment, but providers maintain concerns that patients may be unwilling to participate.

**Methods:**

This mixed-methods study conducted semi-structured interviews of patients presenting to a quaternary care emergency department with acute diverticulitis and a web-based survey of a larger cohort. The interviews assessed patients’ experiences with diverticulitis and perceptions of participation in a trial comparing antibiotics versus placebo. The survey quantified patients’ willingness to participate in such a study and the relative importance of factors influencing the process.

**Results:**

Thirteen patients completed an interview. Reasons to participate included a desire to help others or contribute to scientific knowledge. Doubts about the efficacy of observation as a treatment method were the main barrier to participation. In a survey of 218 subjects, 62% of respondents reported willingness to participate in a randomized clinical trial. “What my doctor thinks,” followed by “What I have experienced in the past” were the most important decision-making factors.

**Conclusion:**

Patients with acute uncomplicated diverticulitis maintain complex and varying perceptions of antibiotic use. Most surveyed patients would be willing to participate in a trial of antibiotics *versus* placebo. Our findings support the trial’s feasibility and may facilitate an informed approach to recruitment and consent.

## INTRODUCTION

Diverticular disease is one of the most prevalent conditions in the United States, and its incidence is increasing. Acute diverticulitis is a leading cause of emergency department (ED) visits, accounting for over 360,000 visits per year.^1^ Despite evidence that antibiotics are not necessary to treat acute uncomplicated diverticulitis (AUD)^2–4^ and models of diverticulitis pathophysiology suggesting an inflammatory process rather than an infectious one,^5,6^ antibiotics remain the mainstay of treatment in the United States.

Unnecessary use of antibiotics is harmful. Antibiotic misuse exacerbates antibiotic resistance, accounting for more dangerous infections and difficult-to-eradicate pathogens. In the United States, over 2.8 million antibiotic-resistant infections develop per year, resulting in over 35,000 associated deaths.^7^ Overuse of antibiotics leads to longer hospital stays, more readmissions, and ultimately higher mortality rates due to infectious disease.^8^ Antimicrobial resistance due to overuse carries a high economic burden of $55 billion per year in the United States.^7^ Management of uncomplicated diverticulitis without antibiotics represents an opportunity to educate prescribers and reduce antibiotic prescriptions for one of the leading causes of ED visits in the United States.

Despite this, antibiotics remain the standard treatment in the United States. Three randomized controlled trials (RCTs) in Europe have demonstrated noninferiority of symptomatic and supportive care without antibiotics compared to treatment with antibiotics for the outcomes of perforation and abscess formation, median hospital stay, and diverticulitis recurrence.^2–4^ Guidelines from the American Gastroenterological Association, American Society of Colon and Rectal Surgeons, and American College of Physicians state that uncomplicated cases can be treated without antibiotics.^9–12^ The reasons for continued antibiotic use are multifactorial, including lack of physician awareness of updated literature and guidelines, physician fear of medicolegal consequences of “observation,” and patient expectation of treatment with antibiotics.^13^ Although 3 RCTs from Europe demonstrated noninferiority of antibiotics, these studies were underpowered for several important secondary outcomes such as progression to complicated diverticulitis, persistent diverticulitis, and sigmoid resection. Additionally, 2 focused on inpatient management, which is not the standard of care in the United States, and the 1 that focused on outpatient management did not include placebo or blinding. A blinded, placebo-controlled trial performed in the United States that is powered for outcomes of interest is needed to demonstrate unequivocal non-inferiority.

The perception that patients expect and desire antibiotics and uncertain evidence of noninferiority of nonantibiotic treatment may prevent providers from assuming this approach. However, the threat of antibiotic resistance is genuine, and the continued inappropriate use of antibiotics is antithetical to a rigorous effort to curtail resistance. A North American RCT of antibiotics *versus* no antibiotics for AUD that demonstrated noninferiority of an antibiotic-free approach would likely help decrease superfluous antimicrobial use for uncomplicated diverticulitis.^13^ This study aims to assess patients’ perceptions and attitudes regarding participation in an RCT of antibiotics *versus* no antibiotics for AUD and evaluate willingness to participate in such a trial.

## MATERIALS AND METHODS

This mixed-methods study assessed patient perspectives through focused interviews followed by a survey of a separate, larger cohort to quantify themes that emerged from the interviews. Vanderbilt University Medical Center (VUMC) Institutional Review Board (IRB) approved this study with the allowance of verbal consent for the focused interviews (IRB #2210530) and a waiver of informed consent for surveys (IRB #221611).

### Focused Interviews

Semi-structured interviews were conducted both in-person in the VUMC ED and over the phone. Individuals eligible to participate in in-person interviews included adults (age 18–90 years), English-speaking patients presenting to the VUMC ED with AUD, and adequate cognitive capacity to participate, as determined by the participant’s ability to accurately recount study activities to the interviewer during the verbal consent process. Exclusion criteria for this group included complicated diverticulitis such as bleeding, abscess, or perforation; asymptomatic diverticulitis found incidentally on computed tomography (CT) scan; history of colon or rectal cancer, Crohn’s disease, or ulcerative colitis; end-stage renal disease; or previous colectomy. Individuals eligible to participate in phone interviews included adult English-speaking patients with a history of AUD treated at VUMC. Eligibility did not require a current episode of AUD, and all phone interviews were conducted with patients with a history of diverticulitis but no current episode. Interviews were conducted from October 2022 to December 2022. Once the study team recognized that no novel themes emerged during interviews, it was determined that thematic saturation was reached, and the survey phase concluded.^14^

The interview guide was created with support from the VUMC Qualitative Research Core.^15^ It was designed to allow patients to reflect on their expectations, experiences, and opinions about diverticulitis using 4 open-ended questions followed by 4 focused questions to obtain thoughts about the side effects of antibiotics, antibiotic resistance, and treatment guidelines (Supplemental Text, http://links.lww.com/AOSO/A393).

Subjects were identified using natural language processing of radiologists’ interpretations of CT scans to identify Unified Medical Language System concepts representing diverticulitis. Study personnel were sent an email alert within minutes of radiology interpretation and reviewed the CT scan, radiology interpretation, and patient chart to ensure eligibility. The interviewer (A.A.-M.), a female medical student who underwent qualitative interview training with the Qualitative Research Core, approached eligible participants and obtained verbal consent for participation. The interviewer had no prior relationship with patients and disclosed that she is a medical student with an interest in the research topic. Interviewees were not compensated. Interviews were recorded using a Sony International Classification of Disease-PX370 digital voice recorder. Phone interview participants were identified through an electronic medical record query of patients seen in the VUMC Colon and Rectal Surgery Clinic. The interviewer called participants and obtained verbal consent for participation.

### Qualitative Analysis

Audio recordings of interviews were transcribed using an online transcription service^16^ and transcripts were verified manually and not shared with participants. Coding and thematic analysis were conducted by A.A.-M. Interview content was analyzed using open coding and thematic analysis. According to the grounded theory approach, theories were derived from raw data by generating and applying codes.^17^ The first 5 transcripts were reviewed, and each line of text was ascribed one or more codes which were derived inductively from the data. After review of the first 5 transcripts, codes were distilled into a codebook which was referenced to code the remaining transcripts. Thematic analysis was conducted through 4 steps of qualitative analysis identified by Green et al^18^—immersion in the data, coding, creating categories, and identification of themes. Qualitative data collection and analysis were performed in accordance with the consolidated criteria for reporting qualitative research (COREQ).^19^

### Survey Methodology

Qualitative analysis of interview data informed the development of a web-based survey to quantify the attitudes and beliefs of a separate, larger cohort. Individuals eligible to participate in surveys included patients with a history of diverticulitis. Exclusion criteria were a history of colon and rectal cancer, Crohn’s disease or ulcerative colitis, end-stage renal disease, or previous colectomy. Study eligibility was based on a medical history submitted via a REDCap presurvey. Participants were given access to the study survey only if they met eligibility based on their self-reported medical history.

Using the coded transcripts, potential survey items reflecting patient opinions about antibiotics and treatment of AUD were written. They were organized into an online survey with 3 sections, each with 5 items that participants were asked to rank from least important to most important. The survey was reviewed and revised by the research team and beta-tested by both physicians and patients (Supplemental Material, http://links.lww.com/AOSO/A394). It was distributed as an online survey using REDCap.^20^ Additional free text items asked participants to describe their reasons for being willing or unwilling to participate in a randomized clinical trial.

Survey participants were identified using social media, VUMC’s electronic health record, and ResearchMatch, a national health volunteer registry supported by the United States National Institutes of Health as part of the Clinical Translational Science Award program. An IRB-approved recruitment message was posted on diverticulitis-related social media pages and sent to eligible ResearchMatch volunteers. Eligible participants identified via International Classification of Disease-10 codes through the electronic health record were contacted with an IRB-approved message through My Health at Vanderbilt, VUMC’s patient health portal (Supplemental Text, http://links.lww.com/AOSO/A395). Participants were given the option to enter a raffle to win a $100 gift certificate upon completion of the survey.

### Survey Data Analysis

Descriptive statistics were performed to examine frequencies, means, and standard deviations in survey data. Ranked factors were viewed by the number of times ranked “most important” and number of times ranked “least important.” A two-tailed, two-sample *T* test assuming unequal variance (Welch’s *T* test) was used to compare the number of previous diverticulitis episodes between those who reported willingness and those who reported unwillingness to participate. Qualitative analysis was applied to participants’ explanations of willingness or unwillingness to participate with an open coding and thematic analysis approach similar to the methods described in the focused interview section.

## RESULTS

### Participant Characteristics

Thirteen patients completed an interview: 9 in the ED with an episode of AUD and 4 over the phone without a current episode (100% participation for in-person interviews, 67% for phone interviews). All were interviewed alone and only once. Most participants were female (62%, n = 8/13) and non-Hispanic White (85%, n = 11/13). Detailed patient characteristics are presented in Table 1. Participants had a mean age of 62 years. Mean interview duration was 16 minutes (SD = 6 minutes).

**TABLE 1.** Characteristics of Interview Participants

Variable		N (%)
Age, mean, (SD)		62.4 (13.0)
Age by category	18–49 years	3 (23)
	50–64 years	5 (38)
	≥65 years	5 (38)
Sex	Female	8 (62)
	Male	5 (38)
Ethnicity	Hispanic or Latino	0 (0)
	Not Hispanic or Latino	13 (100)
Race/Ethnicity	White, Non-Hispanic	11 (85)
	Black or African American	2 (15)
	Asian	0 (0)
	More than one race	0 (0)
Interview type	In-Person	9 (69)
	Phone	4 (31)

### Themes

**FIGURE 1.**
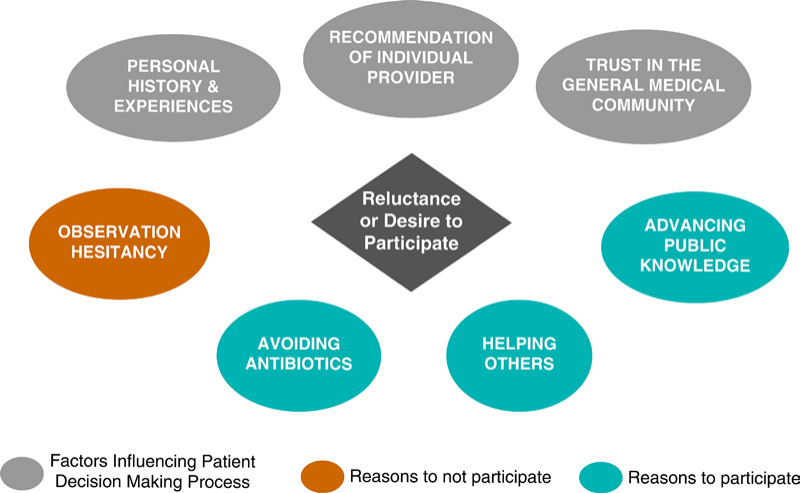
Conceptual model of factors contributing to reluctance or desire to participate.

Two salient themes that emerged were sources of information used in decision-making and factors contributing to reluctance or desire to participate in a trial. Information sources guiding the decision-making process included personal experiences along with provider recommendations. Patients reporting reluctance to participate in a trial commonly expressed doubts about the efficacy of observation as a treatment strategy. Themes regarding willingness to participate were the desire to avoid antibiotics in the future, to help others, and to contribute to public knowledge. Figure 1 demonstrates a conceptual model of factors contributing to reluctance or desire to participate. Table 2 includes quotations illustrating each of these themes.

**TABLE 2.** Summary of Themes and Representative Quotes

Theme	Subtheme	Quotations
Personal history and experiences	Antibiotic effectiveness	With my first episode, I had delayed treatment, so it got extremely infected at a bad abscess…from my experience, catching it as early as possible was critical because delayed treatment clearly, in my experience, made it much worse. (Participant 5).
	Antibiotic inefficacy	I’ve had so many episodes and found that [antibiotics] really didn’t help. And now my interpretation was that I didn’t instantly get better. I got only got better with time as the inflammation settled down. That’s my interpretation. (Participant D).
Provider opinion	Known provider	I would rely on my doctor if he told me that this is a situation that can be alleviated through pain management and some other medication or no medication. (Participant 7).
	ED provider	I guess I’m just kind of going on what [the ED doctor] said. If his recommendation was the antibiotic, then I think that would be what I would do. If he said, you can take this or not, and it’s going to be the same outcome, I don’t know. That might change it if he had said something else. (Participant 6)
	General medical community	I mean, I take it when they tell me I need it and I go blindly…see, as we all do, maybe. (Participant 9).
Observation hesitancy		That would be my number one concern is what if it doesn’t work? How long do I have to stay in pain before we can go back to what works? (Participant 5).
Avoiding antibiotics in the future	Antibiotic hesitancy	I don’t ever like to take an antibiotic unless I have to. Okay. Now I’m taking one drug right now that I… I have to take, but I take it, and it has had side effects. (Participant C).
	Healthcare hesitancy	I used to be involved in the fitness industry and there was a lot of people that were my associates that were very into nutrition, and they believed in healing yourself through healthy eating and exercise and didn’t like medication…. So I think we overdo it and I think we damage our immune systems. (Participant 1).
Public impact	Helping others	I think research is research and as we progress, medicine progresses. So I think whatever needs to be done, for the betterment of everyone, is what needs to be done. (Participant 8)
	Advancing public knowledge	I mean, I want to participate in anything it’s going to help advance treatment. (Participant 2)

### Subtheme 1: Personal History and Experiences

Participants referenced personal experiences of disease episodes when considering participation in a trial and chronicled a recurring sequence of events: diagnosis of diverticulitis, antibiotic administration, and subsequent symptom resolution. Recognizing and reflecting on the pattern of symptom resolution after antibiotics influenced how participants considered participation in a trial where they may not receive antibiotics.

*So to be honest, it seems that from my experience, and I know it’s anecdotal, it seems [to] suggest there was a physiological change from the medication. (Participant 2).*

Participant 2 reflected on his past experiences, and by the end of the discussion, had drawn upon them to reach a decision:

*Well, talking it out, it’s kind of helped me process and see that from a very first episode, it seems like the amoxicillin was effective… So actually, just talking to you, I started to lean more towards staying with amoxicillin. (Participant 2).*

Patients’ perceptions of antibiotic efficacy in the past, whether perceived as high or low efficacy, informed decisions about participation in a trial. This individual perceived that antibiotics were effective during previous episodes, which precipitated reluctance to participate in a trial. Given the recurring and episodic nature of diverticular disease, personal experience is particularly salient to decisions about disease management.

### Subtheme 2: Influence of Providers on Participant Perceptions

Participants considered providers’ recommendations about whether antibiotics would be beneficial as central to their perceptions of diverticulitis treatment. Some participants identified their individual primary care provider or the ED provider as trusted sources of guidance about treatment options.

*I think I would need to talk to my doctor, my family doctor. I think that would be my best bet. (Participant A).*

Others invoked the authority of providers as a collective medical community who have accepted antibiotics as the standard treatment.

*You just trust the science or whatever … it doesn’t matter which doctor, everybody prescribes the antibiotic regardless …. So I’ve never questioned it because that’s what they say it takes. (Participant 5).*

### Subtheme 3: Observation Hesitancy

Most participants who were unwilling to participate in a trial doubted the efficacy of observation as a treatment strategy, believing that antibiotics are necessary for recovery. These participants tended to view the omission of antibiotics as the omission of treatment.

*At least when I have it, I mean, I can’t imagine if there was no treatment, because I’ve had to wait before to get treatment and it didn’t get any better. (Participant 6).*

The interviewer informed participants that the placebo arm would receive treatment in the form of supportive care, pain control, and close observation. However, some participants interpreted the lack of antibiotics as “no treatment.”

*So if I’m getting the sugar pill then I’m in the control group, does that mean I’m not getting any treatment at all? (Participant B).*

### Subtheme 4: Desire to Avoid Antibiotics in the Future

Aversion toward antibiotics contributed to both reluctance and desire to participate. Some patients feared placement in the antibiotic study group and others saw participation in the study as a means of avoiding antibiotics in the future.

*But I never take an antibiotic unless I have to. I just am afraid it would - you take too many and it won’t work. (Participant C*)

Concern about taking any medication and aversion to unnecessary medical treatment in general were commonly shared sentiments.

*Well, I think that people get over-medicated sometimes, I do. (Participant 4*)

### Subtheme 5: Helping Others and Advancing Public Knowledge

The public impact of study participation, including advancing public knowledge and helping others, were primary drivers of patients’ willingness to participate.

*I struggle with this for 10 years and any advancement in the treatment, I’d be glad to be part of that. Yeah, it would help a lot of people, I would think. (Participant 2*)

### Survey Quantitative Results

**FIGURE 2.**
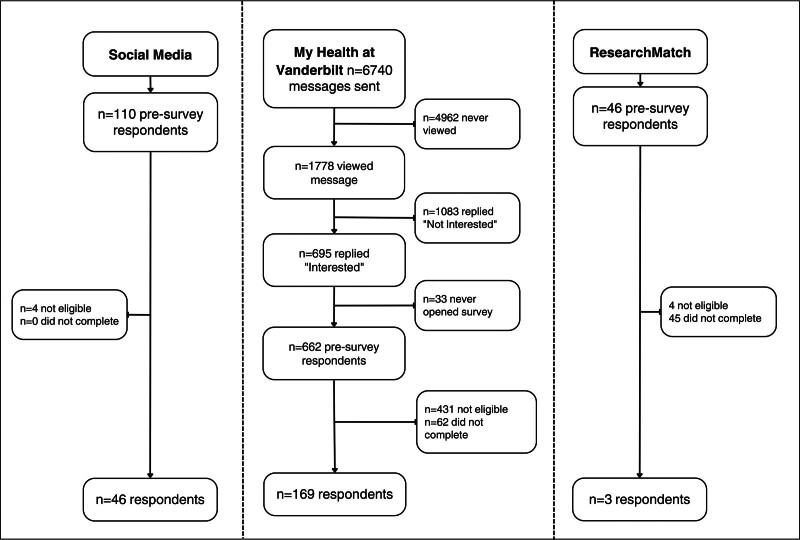
Survey participation rate by recruitment method.

Two hundred and eighteen participants completed the survey. Participation rate by method is demonstrated in Figure 2. Mean participant age was 57.9 years (SD = 13.3 years), and most were female (61.9%), and non-Hispanic White (69.7%). Detailed patient characteristics are presented in Table 3.

**TABLE 3.** Characteristics of Survey Participants

Variable		N (%)
Age, mean, (SD)		57.9 (13.3)
Age by category	18–49 years	58 (26.6)
	50–64 years	68 (31.2)
	≥65 years	81 (37.2)
Sex	Female	135 (61.9)
	Male	77 (35.3)
	Other/prefer not to say	2 (0.9)
	Not reported	4 (1.8)
Ethnicity	Hispanic or Latino	12 (5.5)
	Not Hispanic or Latino	191 (87.6)
	Unknown	3 (1.4)
	Not reported	12 (5.5)
Race	White, non-Hispanic	152 (69.7)
	Black or African American	9 (4.1)
	Asian	1 (0.5)
	More than one race	4 (1.8)
	Not reported	31 (14.2)

**FIGURE 3.**
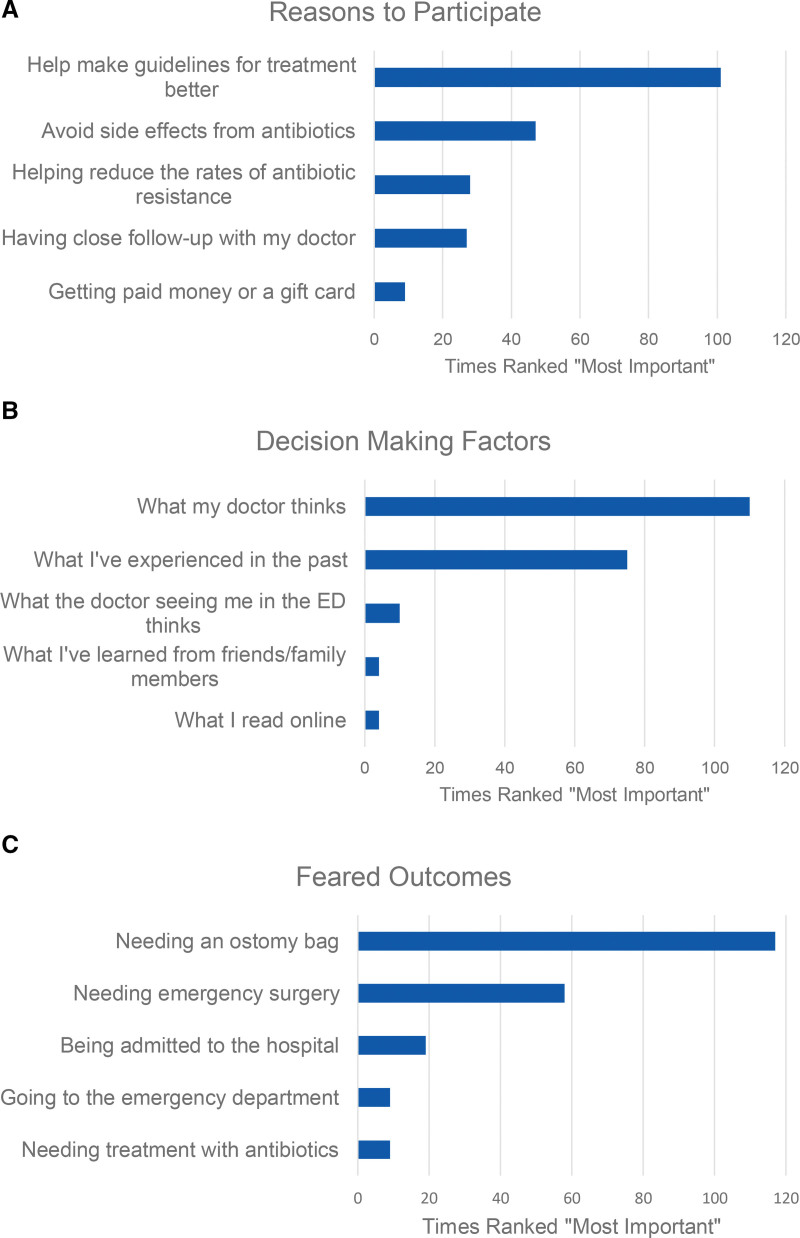
Survey factors represented by times ranked “most important,” including (A), reasons to participate in a randomized controlled trial of antibiotics *versus* placebo, (B), factors used to make healthcare decisions, and (C), feared outcomes of diverticulitis.

One hundred and thirty-five respondents (62%) reported willingness to participate in a trial of antibiotics *versus* placebo during an episode of AUD. Figure 3 illustrates the number of times each factor was ranked most important. The two leading reasons to participate included “helping make guidelines for treatment better” (48%) and “avoiding antibiotics” (22%).

When prompted to rank the importance of various factors used to make healthcare decisions, the leading factors were “What my doctor thinks,” (56%) and “What I have experienced in the past” (36%). Only 5% reported “What the doctor seeing me in the emergency department thinks” as most important. “Needing an ostomy bag” was the most feared outcome of diverticulitis (55%).

There was no significant difference in the number of previous diverticulitis episodes between respondents who were willing *versus* unwilling to participate (*P* = 0.41).

### Qualitative Results of Surveys

Among 83 who reported unwillingness to participate, 67 explained their response. These explanations largely fall into 2 categories: fear of an antibiotic-free treatment and aversion to antibiotics themselves.

*I have had diverticulitis three times and each time it was cleared up with antibiotics. The thought of getting a placebo scares me. (Male in their 60s).*

Fear of not receiving antibiotics contrasts with a preference for a nonantibiotic strategy.

*Don’t trust antibiotics. Makes it worse. Longer to heal. (Male in their 60s).*

Those who were willing to participate specified a desire to help others, contribute to diverticulitis-related research, or understand the disease themselves.

*I like to help and would like a better understanding of diverticulitis. (Female in their 40s).*

## DISCUSSION

Considering that patient hesitancy to participate in a trial of antibiotics *versus* placebo has been a major barrier to its execution, understanding patients’ opinions is essential to conducting a trial and ultimately implementing a change in the standard treatment of diverticulitis. Both qualitative literature and literature regarding patient perspectives are increasingly recognized as important for informing treatment strategies and understanding trial recruitment.^14,21–23^ This mixed-methods study assessed patients’ perceptions of diverticulitis treatment options through semi-structured interviews with patients in addition to a survey of a larger cohort and found that most patients with a history of diverticulitis report willingness to participate in a trial of antibiotics *versus* placebo for AUD. It also identified factors that patients consider when making decisions related to their healthcare, with their doctor’s recommendation being the most important.

Over 60% of survey respondents reported willingness to participate in a trial of antibiotics *versus* placebo for AUD. When compared with participation rates in similar United States surgical trials that have recruited from the ED, this enthusiasm for trial participation would support robust enrollment in an actual trial and scientific integrity. The Comparison of Outcomes of antibiotic Drugs and Appendectomy (CODA) trial, comparing antibiotics with appendectomy for appendicitis, had a 31% enrollment rate among eligible patients.^24^ Our findings support the feasibility of such a trial and can be applied during its planning and execution to facilitate patient-centeredness in methods and analysis. For example, this study highlighted the need to emphasize to potential participants that supportive care includes fluids, pain control, and all other treatment that is standard for AUD except for antibiotics.

Confidence in providers was evident in conversations with patients and survey responses. ED provider opinions carried weight, particularly in interviews, where these providers were the first to communicate diagnoses and present treatment options. Interviewees also expressed confidence in doctors and the medical community to make the best decision for them. These findings highlight the potential for providers to affect patient perspectives on the use of an antibiotic-free strategy.

A key strength of this study is that interviews simulated enrollment in an RCT: most patients were recruited in the ED at the time of diagnosis, before either discharge or hospital admission. This recruitment approach provided a unique view into patient perceptions of treatment options at the time that they would be enrolled and therefore reflected authentic concerns, doubts, and questions that typically arise in that context.

Our study has limitations. There is the possibility of selection bias, which is inherent to conducting a study to evaluate willingness to participate in a study. However, all individuals approached in-person in the ED agreed to participate in an interview, indicating that the population was not skewed toward those who are more inclined to participate. The population sampled was disproportionately white compared with the population affected by diverticulitis, meaning that the perspectives of some voices underrepresented in biomedical research are left out. Another limitation was that survey respondents did not have the opportunity to engage in dynamic discussion with the research team. Some participants misunderstood the explanation of the trial and indicated unwillingness to undergo randomization because they were not currently experiencing symptoms. Consequently, participants’ willingness may have been underestimated. It is also possible that individuals without a history of diverticulitis could have responded to the survey. However, we believe this is unlikely given the modest compensation for study participation and targeted recruitment efforts.

The most important factors guiding patients’ decision-making about diverticulitis treatment include recommendations by trusted providers and perceptions of observation as ineffective treatment. These factors may be modulated over time as providers accept an antibiotic-free approach and affirm its legitimacy to patients. A US-based RCT of antibiotics *versus* placebo for acute uncomplicated diverticulitis is feasible in terms of patient recruitment and even anticipated by patients.

## ACKNOWLEDGMENTS

The authors thank Dario Giuse, Dr. Ing, MS, FACMI, for his contribution to the study.

## AUTHORS CONTRIBUTIONS

A.A.-M. designed and executed all study procedures including manuscript drafting and editing. K.B. assisted interview guide design, qualitative training of the primary author, and manuscript writing. D.S. supported qualitative analysis including the writing of the corresponding manuscript section. J.W. and W.H.S. assisted with interviewee identification through the emergency department and contributed to writing and editing. E.J.G. supported qualitative analysis and contributed to writing and editing. A.T.H. oversaw all study procedures and contributed to writing and editing. All authors reviewed the final manuscript.

## Supplementary Material



## References

[1] Bollom A Austrie J Hirsch W. Emergency department burden of diverticulitis in the USA, 2006-2013. Dig Dis Sci. 2017;62:2694–2703. 28332105 10.1007/s10620-017-4525-y PMC5610055

[2] Chabok A Påhlman L Hjern F; AVOD Study Group. Randomized clinical trial of antibiotics in acute uncomplicated diverticulitis. Br J Surg. 2012;99:532–539. 22290281 10.1002/bjs.8688

[3] Daniels L Ünlü C de Korte N; Dutch Diverticular Disease (3D) Collaborative Study Group. Randomized clinical trial of observational versus antibiotic treatment for a first episode of CT-proven uncomplicated acute diverticulitis. Br J Surg. 2017;104:52–61. 27686365 10.1002/bjs.10309

[4] Mora-López L Ruiz-Edo N Estrada-Ferrer O; DINAMO-study Group. Efficacy and Safety of Nonantibiotic Outpatient Treatment in Mild Acute Diverticulitis (DINAMO-study): a multicentre, randomised, open-label, noninferiority trial. Ann Surg. 2021;274:e435–e442. 34183510 10.1097/SLA.0000000000005031

[5] Piscopo N Ellul P. Diverticular disease: a review on pathophysiology and recent evidence. Ulster Med J. 2020;89:83–88. 33093692 PMC7576390

[6] Zullo A. Medical hypothesis: speculating on the pathogenesis of acute diverticulitis. Ann Gastroenterol. 2018;31:747–749. 30386127 10.20524/aog.2018.0315 PMC6191870

[7] Kadri SS. Key takeaways from the U.S. CDC’s2019 antibiotic resistance threats report for frontline providers. Crit Care Med. 2020;48:939–945. 32282351 10.1097/CCM.0000000000004371 PMC7176261

[8] Dadgostar P. Antimicrobial resistance: implications and costs. Infect Drug Resist. 2019;12:3903–3910. 31908502 10.2147/IDR.S234610 PMC6929930

[9] Peery AF Shaukat A Strate LL. AGA clinical practice update on medical management of colonic diverticulitis: expert review. Gastroenterology. 2021;160:906–911.e1. 33279517 10.1053/j.gastro.2020.09.059 PMC7878331

[10] Stollman N Smalley W Hirano I; AGA Institute Clinical Guidelines Committee. AGA institute clinical guidelines committee. American Gastroenterological Association Institute guideline on the management of acute diverticulitis. Gastroenterology. 2015;149:1944–1949. 26453777 10.1053/j.gastro.2015.10.003

[11] Hall J Hardiman K Lee S; Prepared on behalf of the Clinical Practice Guidelines Committee of the American Society of Colon and Rectal Surgeons. The American Society of Colon and Rectal Surgeons clinical practice guidelines for the treatment of left-sided colonic diverticulitis. Dis Colon Rectum. 2020;63:728–747. 32384404 10.1097/DCR.0000000000001679

[12] Qaseem A Etxeandia-Ikobaltzeta I Lin JS; Clinical Guidelines Committee of the American College of Physicians*. Diagnosis and management of acute left-sided colonic diverticulitis: a clinical guideline from the American College of Physicians [published correction appears in Ann Intern Med. 2023 Apr;176(4):584]. Ann Intern Med. 2022;175:399–415. 35038273 10.7326/M21-2710

[13] Garfinkle R Sabboobeh S Demian M; Management of Uncomplicated Diverticulitis (MUD) Collaborative. Patient and physician preferences for antibiotics in acute uncomplicated diverticulitis: a Delphi consensus process to generate noninferiority margins. Dis Colon Rectum. 2021;64:119–127. 33093297 10.1097/DCR.0000000000001815

[14] Saunders B Sim J Kingstone T. Saturation in qualitative research: exploring its conceptualization and operationalization. Qual Quant. 2018;52:1893–1907. 29937585 10.1007/s11135-017-0574-8 PMC5993836

[15] Vanderbilt University Medical Center (2023). Vanderbilt University Qualitative Research Core. Vanderbilt Institute for Medicine and Public Health. Retrieved May 3, 2023. Available at: https://www.vumc.org/hsr/qualitative-research-core

[16] Chun Tie Y Birks M Francis K. Grounded theory research: a design framework for novice researchers. SAGE Open Med. 2019;7:2050312118822927. 10.1177/2050312118822927 PMC6318722 30637106

[17] Rev Transcription. Available at: https://rev.com. Accessed January 3, 2023.

[18] Green J Willis K Hughes E. Generating best evidence from qualitative research: the role of data analysis. Aust N Z J Public Health. 2007;31:545–550. 18081575 10.1111/j.1753-6405.2007.00141.x

[19] Tong A Sainsbury P Craig J. Consolidated criteria for reporting qualitative research (COREQ): a 32-item checklist for interviews and focus groups. Int J Qual Health Care. 2007;19:349–357. 17872937 10.1093/intqhc/mzm042

[20] Harris PA Taylor R Thielke R. Research electronic data capture (REDCap)--a metadata-driven methodology and workflow process for providing translational research informatics support. J Biomed Inform. 2009;42:377–381. 18929686 10.1016/j.jbi.2008.08.010 PMC2700030

[21] Ndjaboue R Chipenda Dansokho S Boudreault B. Patients’ perspectives on how to improve diabetes care and self-management: qualitative study. BMJ Open. 2020;10:e032762. 10.1136/bmjopen-2019-032762 PMC7213839 32354775

[22] Moorcraft SY Marriott C Peckitt C. Patients’ willingness to participate in clinical trials and their views on aspects of cancer research: results of a prospective patient survey. Trials. 2016;17:17. 26745891 10.1186/s13063-015-1105-3 PMC4706669

[23] Ferrell B Williams AC Borneman T. Clinical trials: understanding patient perspectives and beliefs about treatment. Clin J Oncol Nurs. 2019;23:592–598. 31730601 10.1188/19.CJON.592-598 PMC9059412

[24] Flum DR Davidson GH Monsell SE; CODA Collaborative. A randomized trial comparing antibiotics with appendectomy for appendicitis. N Engl J Med. 2020;383:1907–1919. 33017106 10.1056/NEJMoa2014320

